# 

**Published:** 1902-09-15

**Authors:** 


					﻿THE
DENTAL REGISTER
EDITED BY
NELVILLE S. HOFF, D. D. S.,
ANN ARBOR, MICH.
Vol. LVI. SEPTEMBER i£, 1902.	No. 9.
PRICE, ONE DOLLAR A YEAR IN ADVANCE.
PUBLISHED MONTHLY BY
SAMUEL A. CROCKER & CO.,
THE OHIO DENTAL AND SURGICAL DEPOT.
3“ to :?5> W. “tlx ^t.9 Ciiioimiiiti, O.
Entered at the Postoffice at Cincinnati, 0., as second-class mail matter.
				

